# Association of IL-1 and TNF-αLevels in Endometrial Secretion and Success of Embryo Transfer in IVF/ICSI Cycles

**DOI:** 10.22074/ijfs.2019.5668

**Published:** 2019-07-14

**Authors:** Nayyereh Khadem, Mahsa Mansoori, Matin Attaran, Armin Attaranzadeh, Elnaz Zohdi

**Affiliations:** 1 Department of Obstetrics and Gynecology, Mashhad University of Medical Sciences, Mashhad, Iran; 2 Department of Pathology, Mashhad University of Medical Sciences, Mashhad, Iran

**Keywords:** Embryo Transfer, Endometrium, IL-1/TNF-α, IVF/ICSI, Receptivity

## Abstract

**Background:**

In this work, we have determined the levels of interleukin-1 (IL-1) and tumor necrosis factor-alpha (TNF-α), which function as cytokines in endometrial receptivity, through the endometrial secretion within the eligible individuals and thus studied their relationships with the success or failure of pregnancy in in vitro fertilization/intra cytoplasmic sperm injection (IVF/ICSI) cycles.

**Materials and Methods:**

In this prospective study, 76 women were selected for their first IVF/ICSI and met the study inclusion criteria. All of the patients have undergone the endometrial secretion aspiration prior to performing the oocyte collection. The levels of IL-1and TNF-α were analyzed by the means of enzyme-linked immunosorbent assay method, using special standard kits. The patients were requested to undergo the serum human chorionic gonadotropin measurements and ultrasound evaluation for the purpose of detecting successful implantations and pregnancies.

**Results:**

Among the 76 subjects of the study, 33 (43.4%) patients had a positive beta-human chorionic gonadotropin (β-hCG) and 44 (56.6%) resulted in a negative β-hCG. It should be also noted that through the patients with positive β-hCG, 23 (30.3%) of them displayed fetal heart rate in their transvaginal sonography (TVS). Compared to the group with failed pregnancies and their cytokine levels, we perceived a higher concentration of IL-1 in the group containing successful chemical pregnancies (P=0.00). However, there was no significant difference in terms of clinical pregnancy in the IL-1 levels between the two groups (P=0.06). In addition, there was not any notable difference in the levels of TNF-α between the two groups, neither in terms of chemical nor clinical pregnancy (P=0.8 and P=0.6, respectively).

**Conclusion:**

The current study suggests that higher concentrations of IL-1 in endometrial secretions could be as- sociated with improved endometrial receptivity and IVF outcome. With regards to TNF-α, no statistically significant difference was observed between the groups of with and without successful pregnancies.

## Introduction

Infertility is a common condition which can effect marital relationships, mental health and quality of life of couples (1-3). Recent advances in assisted reproductive techniques (ART), such as in vitro fertilization (IVF) and intra cytoplasmic sperm injection (ICSI), have resulted in development of effective methods for treating infertility. However, these methods are expensive and impose huge costs on families, while a significant number of IVF/ICSI procedures does not result in a live birth (2, 4).

The issue of endometrial preparation is largely overlooked since the infertility clinics often focused on the provision of appropriate quality embryos for transmission. The existing relationship between maternal immune system and embryonic tissues at the time of implantation is considered quiet vital for a successful implantation. This fact has been confirmed by one of the first letters of Betteridge (5). They discussed about the role of endometrial receptivity in the embryo transfer process, while indicating that the existence of an accommodation between the embryo and endometrium is necessary for pregnancy.

Several studies have been conducted to determine the effective factors that seemed to be responsible for the success of ARTs. One of these factors is the group of cytokines, produced by fetus and uterine mucosa. They are responsible for regulating the interaction between mother and fetus, ultimately causing the major influence on improvement of uterus receptivity (6).

Some of the certain known cytokines and growth factors that may contribute to increasing endometrial receptivity include interleukin-1 (IL-1), tumor necrosis factor-alpha (TNF-α), leukemia inhibitory factor (LIF), and transforming growth factor-beta (TGF-ß) (7). For the first time, in 2003, a novel approach on the study of cytokines was presented by van der Gaast et al. (8), evaluating cytokines by analyzing the endometrial secretions. In fact, the endometrial tissue itself is not ideal for assessing biomarkers, due to the complexity of cell, its differentiation between different individuals and even different stages of the cycle. Furthermore, the uterine fluid collection, by either lavage or aspiration, is less invasive than tissue biopsy and ultimately the observed changes in this fluid can be sign of a true microenvironment for implantation. These discoveries have been discussed in detail through a study published by Salamonsen et al. (6). 

Given the prevalence of infertility, high cost of fertility treatments and crucial role of cytokines in the success of these methods, the importance of identifying these cytokines and determining the best time for performing the process of embryo transfer is quiet evident. Therefore, in this study, we have determined the levels of IL-1 and TNF-α in endometrial secretion and assessed their roles in the success of embryo implantation.

## Materials and Methods

### Method of study

This prospective study has been conducted in the Infertility Center of Milad (Mashhad, Iran) from August to December 2017. Subsequent to performing sufficient explanation and signing the informed consent, 79 women enrolled the experiment, with the mean age of 32 years. They were candidates of obtaining their first IVF/ICSI due to tubal factor infertility caused by tubal obstruction (evidence of distal tubal obstruction in hysterosalpingography (HSG) was confirmed by laparoscopy).

The Research and Ethics Committee of Mashhad University of Medical Sciences (Iran) (IR.MUMS. fm.REC.1395.329) approved the study protocol. To determine the factors that are effective on the outcome of IVF, all subjects were provided with the following criteria for enrollment in this study: normal menstrual cycles between 21 and 35 days, less than 40 years of age, BMI less than 30, TSH values less than 10, FSH values less than 10 in the third day of the cycle and antral follicular count (AFC) with at least 5-7 in each ovary on the third day of cycle using the vaginal sonography, normal sperm analysis.

The exclusion criteria were also included: endocrine or metabolic disorders, history of previous pelvic or gynecological surgery (endometriosis, etc.). One or more than one occurrence of previous IVF failure, cigarette smoking, recurrent abortion, OHSS, inappropriate endometrium for embryo transfer (echogenic/non triple line and less than 7 mm), evidence of drosalpinx and uterine anomalies in HSG as well as transvaginal sonography (TVS) and other infertility causes.

### *In vitro* fertilization/intra cytoplasmic sperm injection technique

To stimulate ovulation based on antagonist protocol, on the 3^rd^ day of menstrual cycle, we have subcutaneously injected recombinant-follicle stimulating hormone (FSH) (Gonal-F) with a dosage of 150-225 IU/daily, depending on the AFC of each person. During serial vaginal sonography (using PHILIPS, Affinifi 70W device, Netherlands), after observing at least two follicles above 17 mm and follicular cohorts of 14-16 mm, 10000 U of urinary-human chorionic gonadotropin (hCG) were intramuscularly (IM) injected to induce the final oocyte maturation. Thirty six hours after hCG injection, we have performed the oocyte pick up process.

The luteal phase support was started from the day of pick-up by injection of 50 mg progesterone daily/IM. On the 4^th^ day of progesterone, depending on the embryo grading, we transferred one to three cleavage embryo for each patient through the employment of one infertility specialist, utilizing cook catheter under transabdominal guide. The embryo grades were classified as follow: i. Embryo with the stage-specific cell size, <10% fragmentation and no multi-nucleation, ii. Embryo with stage-specific cell size for the majority of cells, 10-25% fragmentation and no evidence of multi-nucleation, and iii. Embryo with not stage- specific cell size, severe fragmentation (25%) and evidence of multi-nucleation (9) and the individuals conditions (such as the patients age).

The level of serum ß-hCG was checked 14 days after performance of embryo transfer. Upon observing a positive result and an appropriate increase in the titer within 48 hours (confirmation of successful implantation), the patients were subjected to vaginal sonography between 4 and 5 weeks after embryo transfer, to confirm the clinical pregnancy by detecting the gestational sac and fetal heart rate.

### Aspiration and endometrial secretion analysis

Before beginning the pick-up and after washing the perineum and vagina with normal saline, we exposed the cervix through the utilization of a speculum. Subsequent to washing the cervix with normal saline, a mannered catheter was employed to administer 2-3 ml of normal saline into the uterine cavity, using a 2-cc syringe. After 30 seconds of fluid infusion, the fluid was suctioned and transferred into a microtubule. The specimen was inscribed on the micro tube and stored in liquid nitrogen at a temperature of 80°C. This process was completely performed on all of the 76 samples. After collecting the 76 samples within five months, standard kits (IBL, USA) were used to measure IL-1 and TNF-α biomarkers by ELISA method. It should be noted that the safety of this method has been discussed and approved in previous studies.

### Statistical analysis

The results of this study were collected in a coherent manner. After completing the statistical data of the in volved subjects, we performed the statistical analysis through application of SPSSII, version 23 and separately based on chemical pregnancy (positive serum ß-hCG was checked 14 days after embryo transfer and successful implantation was confirmed by the appropriate increase) and clinical pregnancy (observing gestational sac and fetal heart rate (FHR) by TVS, 4-5 weeks after embryo transfer). In this experiment, P<0.05 was considered statistically significant.

## Results

These 79 candidate women for their first IVF/ICSI were enrolled based on the provided criteria. Three cases were excluded, one due to the occurrence of OHSS while the other two contained inappropriate endometrium, followed by freezing their embryos. As the last step, the aspirated endometrial secretions of the enrolled candidates were evaluated for IL-1 and TNF-α mean levels by ELISA method. Findings showed among the 76 patients, 33 of them carried positive ß-hCG (chemical pregnancy) and 43 candidates had negative ß-hCG. Based on positive FHR in TVS (clinical pregnancy) throughout the 76 patients, 23 of them have shown FHR positive while 10 of the have resulted in FHR negative.

There was not any significant statistical difference between these two groups in demographic characteristics including age, body mass index (BMI), duration of infertility, AFC and 3^rd^ day FSH as well as number and grade of transferred embryos, which have been mentioned in details in Table 1.

**Table 1:** Baseline and clinical characterization of pregnant and non-pregnant groups


Characteristic		β-hCG^+^n=33	β-hCG^-^ n=43	P value

Age (Y)		33.3 ± 5.3	32.8 ± 5.3	0.7
Duration of infertility		6 ± 4.2	7.3 ± 4	0.1
Number of transferred embryos		1.8 ± 0.4	1.8 ± 5.4	0.7
Grade of transferred embryos				
	A^a^	25 (75.7)	33 (76.7)	0.1
	B^b^	8 (24.2)	10 (23.2)	0.1
BMI		24.6 ± 3.7	23.8 ± 3.8	0.1
Day 3 FSH (IU/L)		8.3 ± 5.4	7.8 ± 3.9	0.2
AFC		12 ± 6.2	11 ± 6.4	0.1


Data are presented as mean ± SD or n (%). a; Embryo with stage-specific cell size, <10% fragmentation and no multi-nucleation, b; Embryo with stage-specific cell size for the majority of cells, 10-25% fragmentation and no evidence of multi-nucleation, ß-hCG; Beta- human chorionic gonadotropin, BMI; Body mass index, FSH; Follicular stimulating hormone, and AFC; Antral follicular count.

In terms of IL-1, chemical pregnancy (positive ß-hCG) group significantly carrier higher level than that of the negative ß-hCG group (P=0.000, Table 2). Although there were higher levels of IL-1 in FHR positive group in terms of clinical pregnancy (observing FHR in TVS), yet the difference has not been statistically notable (P=0.06, Table 3). 

**Table 2:** Comparison of IL-1β and TNF-α levels in aspirated endometrial secretions in patients with positive and negative value of chemical pregnancy


Characteristic	β-hCG^+^Median (25-75)	β-hCG^-^	P value

TNFα (ng/dL)	6 (3.6-7)	5.6 (3.6-7.6)	0.8
IL-1 (ng/dL)	11.4 (2.8-34.2)	2.4 (1-4)	0.000


IL-1ß; Interleukin-1 beta, TNF-α; Tumor necrosis factor-alpha, and ß-hCG; Beta-human chorionic gonadotropin.

**Table 3:** Comparison of IL-1 and TNF-α levels in aspirated endometrial secretions in patients with positive and negative value of clinical pregnancy


Characteristic	FHR^+^n=23 Median (25-75)	FHR^-^ n=10	P value

TNF-α (ng/dL)	4.6 (3.3-7.1)	5.8 (3.6-7.4)	0.6
IL-1 (ng/dL)	5.3 (1.9-15.9)	2.6 (1.1-7)	0.06


IL-1ß; Interleukin-1 beta, TNF-α; Tumor necrosis factor-alpha, and FHR; Fetal heart rate.

## Discussion

Several studies have confirmed the role of interaction between embryo-endometrium and cytokines in implantation. To be stated as an example, in a study published by the Journal of Reproductive Biology in 2009, Haouzi et al. (10) reported that successful implantation of fetus is highly dependent on the fetus quality and endometrial reception.

Nieuwenhuizen et al. (11) and Lieberman et al. (12) mentioned in their reviews that cytokines, which are produced by fetuses and mucous membranes, are responsible for improving the endometrial receptivity. It has also been noted in a study by Zhou et al. (13), that IL-1 stands as an important factor through interaction between embryo and endometrium, as a functional factor during implantation in both maternal and fetal sites. This particular review was confirmed by other studies, such as Bourdiec and Akoum (14), pointing out the effective role of IL-1 through the success of embryo implantation process.

In accordance with the study done by Sequeira et al. (15) the levels of IL-1 in maternal serum levels and median culture of developing embryos were significantly higher in women with successful implantation. Moreover, a study by Loetscher et al. (16) has stated that TNF-α was at a very high level in people with a history of recurrent abortion and infertility.

Aside from this fact, Reid et al. (17) has also discovered that TNF-α is apparently associated with infertility and recurrent abortion, which can be considered as a confirmation of Clark’s study. However, in contrast to Clark and Reid studies, a review performed by Boomsma et al. (18) from Netherlands in 2009, exhibited the existence of a positive correlation between successful pregnancy and higher levels of TNF-α and lower levels of IL-1 in endometrial secretions.

Similar to the present work, Rahiminejad et al. (1), assessed the levels of IL-1 and TNF-α in the endometrial fluid along with their effects on implantation success. They concluded that lower levels of TNF-α in endometrial secretions results in the improvement of endometrial reception. However, no significant difference between IL-1 of the two groups was observed in terms of increasing chance of performing successful implantation.

At the end, next to the contradictions that are theoretically related to the relationship between the levels of these cytokines and successful outcome of pregnancy, various limitations such as technical differences in the aspiration-discharge procedure, aspiration scheduling, low sample size, the leading cause of tubal factor and the confounding effect of drugs could be the cause of the existing differences between the obtained results of different studies and the present investigation. In fact, evaluating the endometrial receptivity probably stands as one of the next steps that will be taken in infertility clinics. This particular test can provide clear information about the inferiority of endometrial receptivity as a primary cause of infertility and ultimately, contribute to the probable prediction of embryo transfer outcome in IVF/ICSI cycles. 

The present study has concluded that there is not any significant statistical relationship between higher levels of IL-1 in endometrial secretion and successful implantation in IVF/ICSI cycles. Furthermore, with regards to the case of TNF-α, we have not discovered any statistical significant difference between the two groups with successful and unsuccessful implantation.

We believe that it would be quiet useful if researchers investigate some other categories, such as association between cytokine levels and ongoing pregnancy rate or even live birth rate, in addition to the relationship between cytokine levels and successful implantation with any specific infertility etiology.

## Conclusion

This study suggests that higher level of IL-1 in endometrial secretions may associate with improved endometrial receptivity and subsequently, this can be related to the improved IVF/ICSI outcomes. In fact, this noninvasive method can enhance the understanding of immunological events, which are involved in the implantation process of fetus. 
